# The KRAS-G12D mutation induces metabolic vulnerability in B-cell acute lymphoblastic leukemia

**DOI:** 10.1016/j.isci.2022.103881

**Published:** 2022-02-07

**Authors:** Yan Xu, Houshun Fang, Yao Chen, Yabin Tang, Huiying Sun, Ziqing Kong, Fan Yang, Renate Kirschner-Schwabe, Liang Zhu, Alex Toker, Ning Xiao, Bin-Bing S. Zhou, Hui Li

**Affiliations:** 1 Key Laboratory of Pediatric Hematology and Oncology Ministry of Health, Pediatric Translational Medicine Institute, Shanghai Children's Medical Center, School of Medicine, Shanghai Jiao Tong University, Shanghai, China; 2 Fujian Children’s Hospital, Fujian Branch of Shanghai Children's Medical Center Affiliated to Shanghai Jiaotong University School of Medicine, Fujian, China; 3 Department of Pharmacology and Chemical Biology, School of Basic Medicine and Collaborative Innovation Center for Translational Medicine, Shanghai Jiao Tong University School of Medicine, Shanghai, China; 4 Hangzhou Calibra Diagnostic, Ltd, Hangzhou, China; 5 Charité Universitätsmedizin Berlin, Berlin, Germany; 6 German Cancer Consortium and German Cancer Research Center, Heidelberg, Germany; 7 Beth Israel Deaconess Medical Center, Harvard Medical School, Boston, MA 02115, USA; 8 Clinical Research Center, Longhua Hospital, Shanghai University of Traditional Chinese Medicine, Shanghai, China

**Keywords:** Biological sciences, Biochemistry, Molecular biology

## Abstract

Mutations in RAS pathway genes are highly prevalent in acute lymphoblastic leukemia (ALL). However, the effects of RAS mutations on ALL cell growth have not been experimentally characterized, and effective RAS-targeting therapies are being sought after. Here, we found that Reh ALL cells bearing the KRAS-G12D mutation showed increased proliferation rates *in vitro* but displayed severely compromised growth in mice. Exploring this divergence, proliferation assays with multiple ALL cell lines revealed that the KRAS-G12D rewired methionine and arginine metabolism. Isotope tracing results showed that KRAS-G12D promotes catabolism of methionine and arginine to support anabolism of polyamines and proline, respectively. Chemical inhibition of polyamine biosynthesis selectively killed KRAS-G12D B-ALL cells. Finally, chemically inhibiting AKT/mTOR signaling abrogated the altered amino acid metabolism and strongly promoted the *in vivo* growth of KRAS-G12D cells in B-ALL xenograft. Our study thus illustrates how hyperactivated AKT/mTOR signaling exerts distinct impacts on hematological malignancies vs. solid tumors.

## Highlights

• Reh ALL cells bearing KRAS-G12D displayed compromised growth in mice
• KRAS-G12D rewires methionine and arginine metabolism in B-ALL cells
• Chemically inhibiting polyamine biosynthesis can potently kill KRAS-G12D ALL cells
• Moderate mTOR inhibition can rescue *in vivo* growth of KRAS-G12D B-ALL cells

## Graphical abstract

**Figure:**
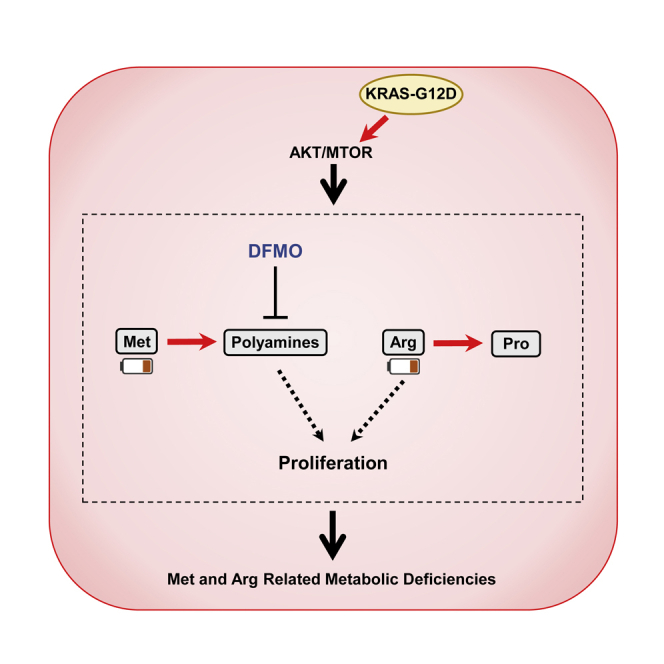

## Introduction

Mutations in RAS pathway proteins (*e.g.*, *KRAS*, *NRAS*, *FLT3*, *PTPN11*, and *NF1*) are highly prevalent in acute lymphoblastic leukemia (ALL), with such mutations occurring in 40%–50% of pediatric B cell ALL cases (Irving et al., 2014; Jerchel et al., 2018; Oshima et al., 2016; Zhang et al., 2011). Most recurrent variants involve activating mutations of *KRAS* and *NRAS*, and glycine residues 12 and 13 (G12/13) are the most common mutation sites in RAS proteins in ALL (Li et al., 2020; Zhang et al., 2011).

It is known that RAS proteins can drive metabolic reprogramming to support rapid tumor growth (Najumudeen et al., 2021; Son et al., 2013; Ying et al., 2012), for example, leading to increased cellular demand for glucose and glutamine in pancreatic cancer (Son et al., 2013). There are several studies of solid tumors showing that oncogenic RAS can reprogram glucose metabolism in tumor cells to support rapid proliferation, increase glutamine utilization to provide extra energy, and enhance cysteine uptake to reduce oxidative stress (Hu et al., 2020; Najumudeen et al., 2021; Son et al., 2013; Ying et al., 2012; Yun et al., 2009). However, the potential relevance of these findings to leukemia remains to be examined. Indeed, there have been very few experimental studies about how RAS mutations affect the growth of cells involved in hematological malignancies.

In the present study, we employed the ALL cell lines Reh and BaF3 to examine the effects of KRAS-G12D, a common RAS mutation, on B-ALL cell growth. After initially observing a profound reduction in the proliferation rate of KRAS-G12D mutation-bearing cells in the bone marrow of Reh xenografts, we discovered that these cells were not selectively affected by altering glucose but were particularly sensitive to limiting extracellular concentrations of various amino acids. We subsequently characterized a dramatic metabolic rewiring in B-ALL cells expressing KRAS-G12D based on metabolite profiling and isotope tracing: these cells display dramatically elevated catabolism of methionine and arginine and show corresponding increases in polyamine and proline biosynthesis. We then harnessed these metabolic insights for translational development, successfully demonstrating that targeting a polyamine biosynthesis enzyme with chemical inhibitors can severely restrict proliferation of KRAS-G12D B-ALL cells. Moreover, our finding that chemical inhibition of mTOR promotes strong *in vivo* proliferation of KRAS-G12D B-ALL cells clearly differentiates the impacts of hyperactivated AKT/mTOR signaling in *RAS* mutant hematological malignancies from previous impacts reported for *RAS* mutant solid carcinomas.

## Results

### B-ALL cells expressing the KRAS-G12D mutant display compromised growth under nutrient-limited conditions

**Figure 1:**
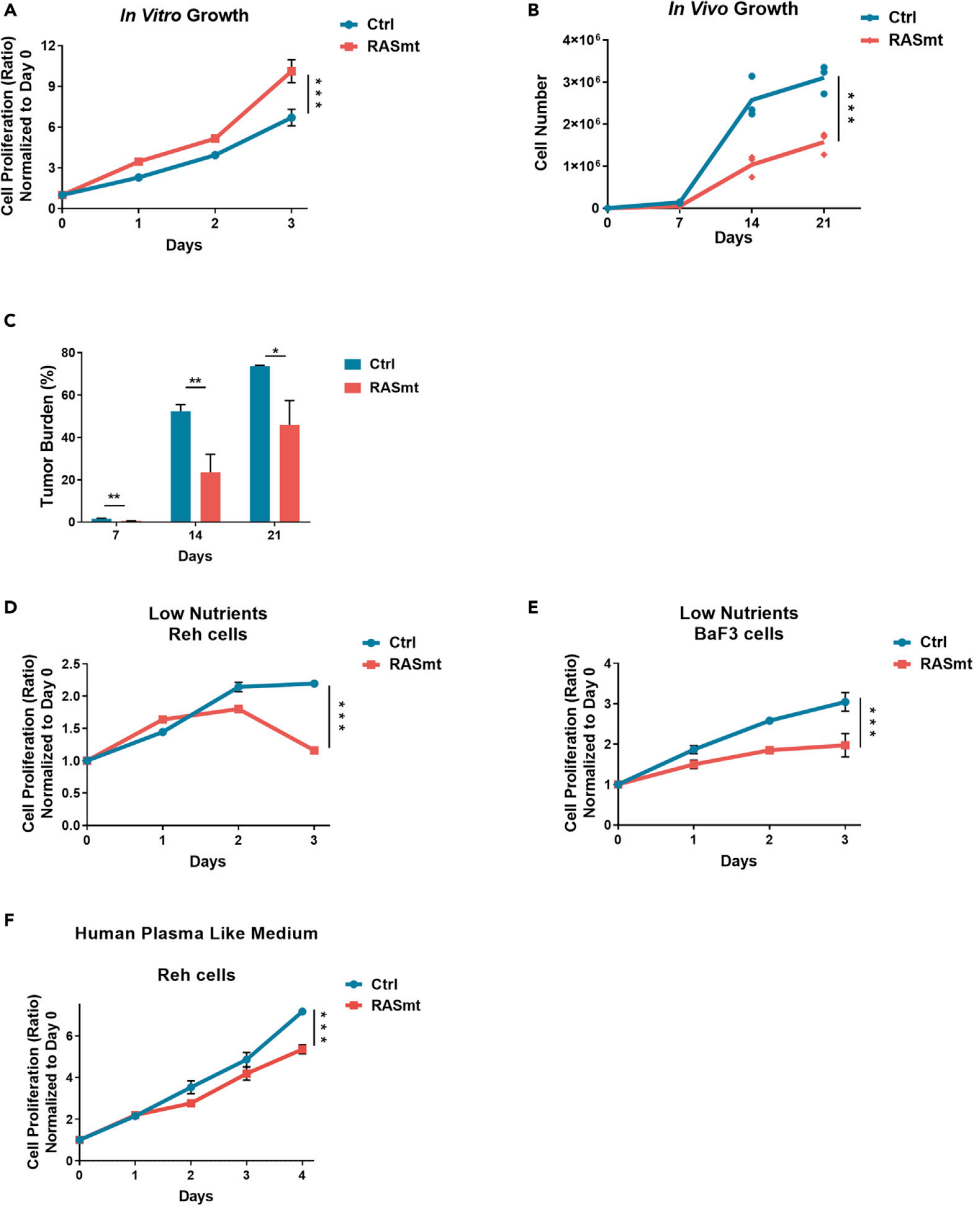
B-ALL cells expressing the KRAS-G12D mutant display compromised growth under nutrient-limited conditions (A) Growth curves of various Reh cells cultured in normal RPMI 1640 medium (as the “nutrient-proficient” condition). Data are shown as mean ± SD ∗∗∗: p < 0.005; two-tailed Student’s *t*-tests. Ctrl: control Reh cells, RASmt: Reh cells expressing KRAS-G12D. (B) The *in vivo* growth kinetics of various Reh xenograft cells. Cells were isolated from xenograft tibias and counted at different time points (day 7, 14, and 21) after tail-vein injection; at each time point, n = 3, the numbers of GFP^+^ Reh cells were determined by flow cytometry. Data are shown as the mean. ∗: p < 0.05, ∗∗∗: p < 0.005; two-tailed Student’s t-tests. (C) The tumor burden of various Reh xenografts (the percentage of GFP-labeled Reh cells in total bone marrow cells) at the indicated time points after tail-vein injection; at each time point, n = 3. Data are shown as the mean ± SD ∗∗: p < 0.01, ∗: p < 0.05; two-tailed Student’s *t*-tests. (D) Growth curves of various Reh cells cultured in RPMI 1640 medium containing low concentrations of nutrients (1 mM glucose and 30-fold diluted concentrations of amino acids); the “nutrient-limited” condition. Data are shown as the mean ± SD ∗∗∗: p < 0.005; two-tailed Student’s *t*-tests. (E) Growth curves of various BaF3 cells cultured in RPMI 1640 medium containing low concentrations of nutrients (1 mM glucose and 30-fold diluted concentrations of amino acids) and murine IL-3 (0.1 ng/mL); the “nutrient-limited” condition. Data are shown as the mean ± SD ∗∗∗: p < 0.005; two-tailed Student’s *t*-tests. Ctrl: control BaF3 cells, RASmt: BaF3 cells expressing KRAS-G12D.F. Growth curves of various Reh cells cultured in HPLM medium. Data are shown as mean ± SD ∗∗∗: p < 0.005; two-tailed Student’s t-tests. Ctrl: control Reh cells, RASmt: Reh cells expressing KRAS-G12D. (F) Growth curves of various Reh cells cultured in HPLM medium. Data are shown as mean ± s.d. ∗∗∗: p<0.005; two-tailed Student’s t-tests. Ctrl: control Reh cells, RASmt: Reh cells expressing KRAS-G12D.

To explore the potential impacts of RAS pathway mutations in B-ALL, we expressed a canonical hotspot KRAS-G12D mutation (henceforth referred to “RASmt”) or an empty vector (“Ctrl” in text) in the human B-ALL cell line Reh. The RASmt cells had a relatively higher proliferation rate than the control Reh cells (Figure 1A). We attempted to further verify this result *in vivo* by using xenograft models, but the growth rate of KRAS-G12D mutant cells *in vivo* was much reduced compared to the Ctrl cells (Figures 1B and 1C). This is not entirely unexpected, as a culture medium is not consistent with the plasma or tissue environment in animals (*e.g.*, in terms of the types and concentration of various biomolecules) (Behrmann et al., 2018; Cantor et al., 2017).

We reasoned that altered levels of some nutrients may help explain the observed differences in the *in vitro* vs. *in vivo* growth of KRAS-G12D mutant Reh cells. Pursuing this, we grew Ctrl and RASmt cells in culture media containing low nutrients (RPMI 1640, with low glucose and amino acids, defined as the “low nutrient” condition) and found that the growth of KRAS-G12D mutant cells was compromised, similar to our findings with the Reh xenograft model *in vivo* (Figure 1D). Notably, we observed a similar growth trend when we assessed another pro-B cell line (BaF3) (Figure 1E). To recapitulate the *in vivo* situation more faithfully, we seeded Ctrl and RASmt Reh cells in the human plasma-like medium (HPLM), whose formulation is consistent with that of human plasma (Cantor et al., 2017) and found that KRAS-G12D compromised the growth of Reh cells (Figure 1F). Together, these results suggested that the KRAS-G12D mutation somehow alters the dependence of B-ALL cells on some nutrient(s) that may be limiting factors for continuous B-ALL cell growth.

### The KRAS-G12D mutation sensitizes B-ALL cells to extracellular amino acids

**Figure 2:**
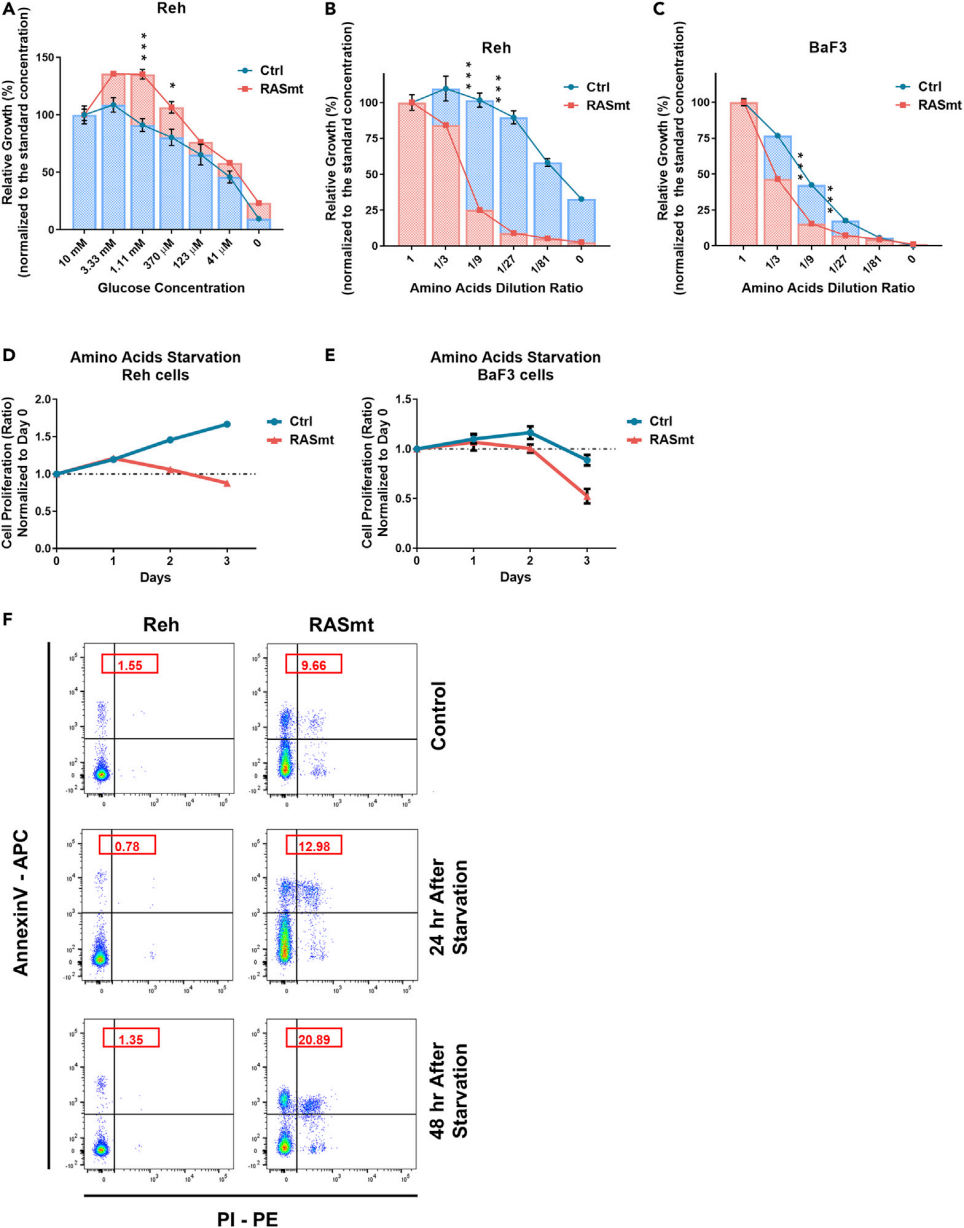
The KRAS-G12D mutation sensitizes B-ALL cells to extracellular amino acids (A) The viabilities of various Reh cells grown in media containing different concentrations of glucose for 72 h. 10 mM is the standard glucose concentration in normal RPMI 1640 medium. Data are shown as the mean ± SD ns: p > 0.05, ∗: p < 0.05, ∗∗∗: p < 0.005; two-tailed Student’s *t*-tests. Ctrl: control Reh cells, RASmt: Reh cells expressing KRAS-G12D. (B and C) The viabilities of various Reh (B) or BaF3 (C) cells grown in media with different dilution ratios of a total 20 amino acids mixture for 72 h. BaF3 cells were cultured in media with a low concentration of IL-3 (0.1 ng/mL) for 72 h. Data are shown as the mean ± SD ∗∗: p < 0.01, ∗∗∗: p < 0.005; two-tailed Student’s *t*-tests. Ctrl: control cells, RASmt: cells expressing KRAS-G12D. (D) Growth curves for various Reh cells cultured in amino-acid-free RPMI 1640 medium. Data are shown as the mean ± SD. (E) Growth curves of various BaF3 cells cultured in amino-acid-free RPMI 1640 medium. Data are shown as the mean ± SD. (F) Apoptosis levels of various Reh cells with or without total amino acid starvation for the indicated times. The apoptosis levels were determined by Annexin V and PI staining; the numbers represent the proportions of Annexin V positive cells. See also Figure S1.

Recalling that RASmt cells showed compromised growth under the “low nutrient” condition (low glucose and amino acids) *in vitro*, we first assessed the effects of glucose. Altering the glucose level did not result in lower relative growth rate of the RASmt cells than Ctrl cells (Figure 2A), suggesting that glucose is not a limiting factor for the growth of B-ALL cells bearing the KRAS-G12D mutation. We then explored impacts of amino acids on KRAS-G12D B-ALL cell growth and found that the relative growth rate of RASmt B-ALL cells was more sensitive to altered concentrations of extracellular amino acids (Figures 2B and 2C). We also determined effects of amino acids on ALL cells with endogenous KRAS mutations (CEM and KOPN8) (Tate et al., 2019) and observed that the relative growth rates of these cells were also more sensitive to concentration changes of extracellular amino acids in comparison with Reh cells (with endogenous wild-type KRAS) (Figure S1). Moreover, total amino acid starvation had a much more pronounced anti-proliferative effect on RASmt Reh cells than on Ctrl cells, and a similar albeit less severe trend was observed with RASmt and Ctrl BaF3 cells (Figures 2D and 2E). We also found that amino acid starvation elicited a greater extent of apoptosis in RASmt cells than in control cells (Figure 2F). These results support that the KRAS-G12D mutation somehow alters the capacity of B-ALL cells to take up and/or metabolize amino acids from the extracellular environment.

### Met and Arg deprivation retard the growth of the KRAS-G12D B-ALL cells

**Figure 3:**
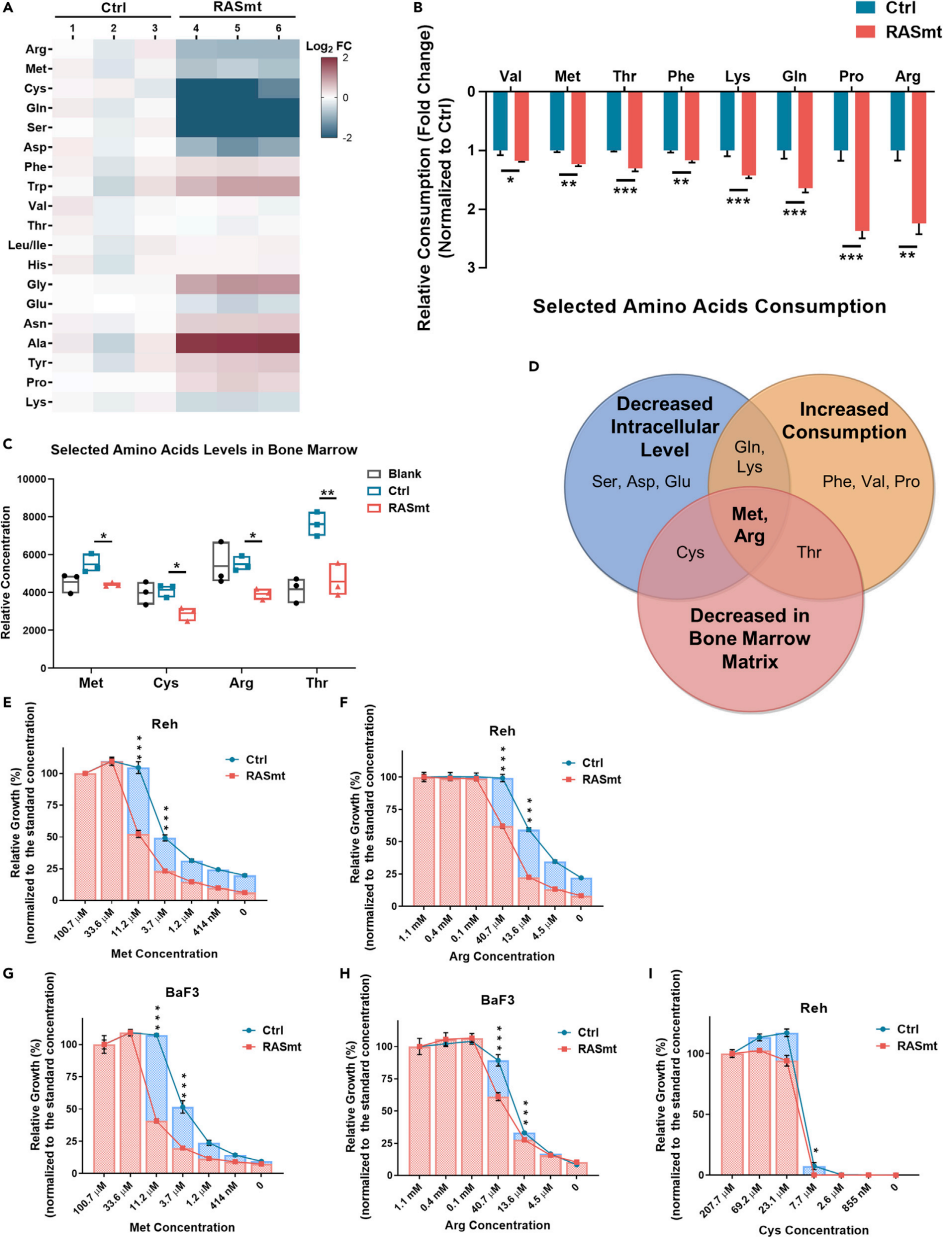
Growth of KRAS-G12D B-ALL cells can be decreased by Met and Arg deprivation (A) Heatmap indicating the fold changes (FC) for the intracellular levels of the indicated amino acids in various Reh cells grown in normal media. Ctrl: control Reh cells, RASmt: Reh cells expressing KRAS-G12D. (B) “Consumption levels” of various Reh cells grown in “normal condition” medium; the consumption level was determined as difference between each amino acid concentration in the medium with Reh cells and the concentration in the medium without cells after 24 h culture. All amino acids with increased consumption in RASmt cells are shown. Data are shown as the mean ± SD ns: p > 0.05, ∗: p < 0.05, ∗∗: p < 0.01, ∗∗∗: p < 0.005; two-tailed Student’s *t*-tests. (C) The relative concentrations of the indicated amino acids in mouse bone marrow matrix solutions, extracted from tibias and femurs of various xenografts, n = 3. The cell-free bone marrow matrix was extracted when the tumor burdens of xenografts are approximately equal (25%–35%). All amino acids with decreased levels in bone marrow of mice injected with RAS-mutant cells are shown. Data are shown as the mean ± SD ∗: p < 0.05, ∗∗: p < 0.01, ∗∗∗: p < 0.005; two-tailed Student’s *t*-tests. Blank: mice without Reh cells injection, Ctrl: mice injected with control Reh cells, RASmt: mice injected with RAS-mutant cells. (D) A Venn diagram indicating the overlap for results from Figures 3A–3C. (E) The viabilities of various Reh cells grown in medium with different concentrations of Met for 72 h. 100.7 μM is the standard concentration of Met in the normal RPMI 1640 medium. Data are shown as the mean ± SD ∗∗∗: p < 0.005; two-tailed Student’s *t*-tests. (F) The viabilities of various Reh cells grown in medium with different concentrations of Arg for 72 h. 1.1 mM is the standard concentration of Arg in normal RPMI 1640 medium. Data are shown as the mean ± SD ∗∗∗: p < 0.005; two-tailed Student’s *t*-tests. (G) The viabilities of the various BaF3 cells grown in media with different concentrations of Met for 72 h. BaF3 cells were cultured in media with a low concentration of IL-3 (0.1 ng/mL). 100.7 μM is the standard concentration of Met in normal RPMI 1640 medium. Data are shown as the mean ± SD ∗∗∗: p < 0.005; two-tailed Student’s *t*-tests. (H) The viabilities of various BaF3 cells grown in media with different concentrations of Arg for 72 h. BaF3 cells were cultured in media with a low concentration of IL-3 (0.1 ng/mL). 1.1 mM is the standard concentration of Arg in normal RPMI-1640 medium. Data are shown as the mean ± SD ∗∗∗: p < 0.005; two-tailed Student’s *t*-tests. Ctrl: control BaF3 cells, RASmt: BaF3 cells expressing KRAS-G12D. (I) The viabilities of various Reh cells grown in medium with different concentrations of Cys for 72 hr. 207.7 μM is the standard concentration of Cys in the normal RPMI 1640 medium. Data are shown as the mean ± s.d. ∗: p<0.05; two-tailed Student’s t-tests.See also Figure S2.

To determine which specific amino acid(s) may contributed to the observed compromised growth of KRAS-G12D mutant B-ALL cells, we profiled the intracellular levels of amino acids in Reh cells cultured in normal medium and found that RASmt cells had significantly reduced levels of arginine (Arg), methionine (Met), serine (Ser), cysteine (Cys), and glutamine (Gln) compared to control cells (Figure 3A). We also measured the changes of amino acids levels in the culture medium and calculated the consumption level of each amino acid in Reh cells (determined as difference between each amino acid concentration in the medium with Reh cells and the concentration in the medium without cells after 24 h culture). Eight amino acids were consumed more rapidly in RASmt cells than in Ctrl cells (Figure 3B). We also examined the levels of individual amino acids in cell-free extracts from the bone marrow matrix of tibias and femurs of Reh xenograft model mice. The levels of Arg, Met, Cys, and Thr (threonine) were significantly decreased in RASmt xenograft samples than in Ctrl animals (Figure 3C).

Notably, both the intracellular and extracellular levels of Met and Arg decreased synchronously in the RASmt setting (Figure 3D). We therefore cultured RASmt and Ctrl cells in various media and ultimately found that deficiency for Met and Arg both obviously impacted the relative growth rate of KRAS-G12D mutant cells compared to Ctrls (for both Reh and BaF3 cells) (Figures 3E–3H); no such difference in the relative growth rate was observed in media deficient for cysteine (Figure 3I). Furthermore, the relative growth rates of ALL cells with endogenous KRAS mutations (CEM and KOPN8) were also affected more severely by altered concentrations of extracellular Met and Arg than that of Reh cells (with endogenous wild-type KRAS) (Figure S2A). To confirm the role of Met and Arg in the cell growth of KRAS-G12D cells, we supplied RASmt Reh cells with Met and Arg under amino-acids-deprived condition and observed that Met and Arg supplementation could partially rescue the relative growth rate of RASmt cells (Figure S2B). Thus, cells bearing the KRAS-G12D mutation are vulnerable to Met and Arg deficiency.

### KRAS-G12D increased Met and Arg catabolism to support the anabolism of polyamines and proline respectively in B-ALL cells

**Figure 4:**
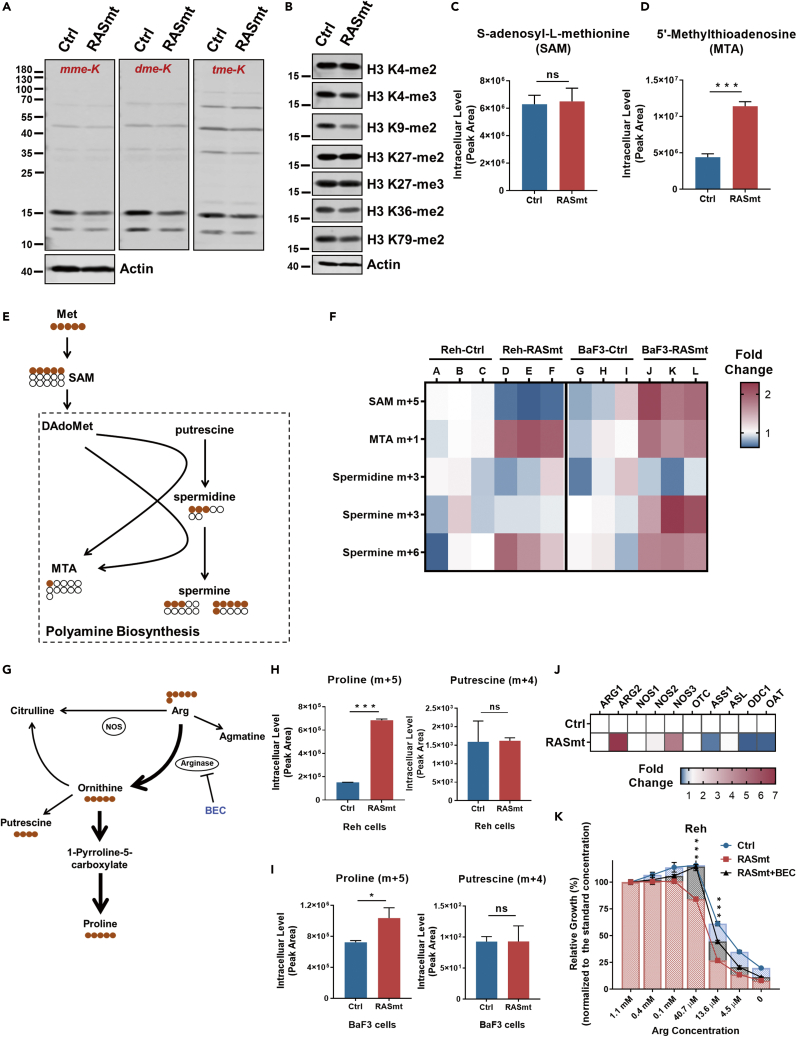
KRAS-G12D increased Met and Arg catabolism to support anabolism of polyamines and proline, respectively, in B-ALL cells (A) Immunoblotting with antibodies against monomethyl lysine (mme-K), di-methyl lysine (dme-K), and tri-methyl lysine (tme-K) of the methylation levels of total proteins in control (Ctrl) Reh cells or Reh cells expressing KRAS-G12D (RASmt). (B) Immunoblot showing methylation levels of H3 histones in Ctrl and RASmt Reh cells. (C) The intracellular levels of S-adenosyl-L-methionine (SAM; a downstream metabolite of methionine) in Ctrl and RASmt Reh cells. Data are shown as the mean ± SD ns: p > 0.05; two-tailed Student’s t-tests. (D) The intracellular levels of 5′-methylthioadenosin (MTA; another downstream metabolite of methionine) in Ctrl and RASmt Reh cells. Data are shown as the mean ± SD ∗∗∗: p < 0.005; two-tailed Student’s t-tests. (E) A methionine metabolism schematic related to the isotope tracing experiment using ^13^C_5_-Met and assessing polyamine biosynthesis. (F) A heatmap indicating fold changes in the intracellular levels of labeled metabolites after ^13^C_5_-Met incubation for 4 h. (G) A schematic for isotope tracing with ^13^C_6_-Arg to assess proline biosynthesis. (H and I) The intracellular levels of labeled proline and putrescine in Reh cells (H) or BaF3 cells (I) after ^13^C_6_-Arg incubation for 5h. Data are shown as the mean ± SD ∗: p < 0.05, ∗∗∗: p < 0.005, ns: p > 0.05; two-tailed Student’s *t*-tests. (J) A heatmap indicating fold changes in relative expression of key enzyme genes associated with Arg metabolism in indicated Reh cells according to the quantification by real-time PCR. (K) The viabilities of Reh cells pre-treated (or untreated) with the known arginase inhibitor BEC (1 mM) and grown in media with different concentrations of Arg for 72 h. 1.1 mM is the standard concentration of Arg in normal RPMI 1640 media. Data are shown as the mean ± SD ∗∗∗: p < 0.005 (RASmt vs. RASmt + BEC); two-tailed Student’s *t*-tests. See also Figure S3.

Downstream metabolites from Met are required for to support elevated protein methylation (Wang et al., 2019b); nevertheless, we detected no increase of total protein or histone H3 methylation in RASmt cells compared to Ctrl cells when they were growing in normal medium (Figures 4A and 4B), nor were there differences in the intercellular levels of the methyl group donor S-adenosylmethionine (SAM) (Figure 4C). However, we did detect significant accumulation of 5′-methylthioadenosine (MTA)—a polyamine biosynthesis intermediate produced from SAM—in the RASmt cells grown in normal medium (Figure 4D). Further, we conducted isotope tracing experiments using ^13^C-labeled Met, which confirmed the enhancement of MTA biosynthesis in RASmt cells (both Reh and BaF3 cells; Figures 4E and 4F).

We also performed isotope tracing with ^13^C-labeled Arg in Reh and BaF3 cells and observed enhanced proline anabolism from Arg in KRAS-G12D cells (Figures 4G–4I), consistent with the aberrantly accumulated proline we earlier observed in RASmt B-ALL cells (Figure 3A). However, we did not find any increase in putrescine biosynthesis from Arg (Figures 4G–4I), suggesting that the increase of polyamine anabolism we observed in KRAS-G12D cells might be supplied by other amino acids (e.g., glutamine) (Li et al., 2016; Puleston et al., 2021) (Figure S3), not Arg. Furthermore, we noticed an increased expression of arginase-2 (ARG2) in KRAS-G12D cells (Figure 4J), which can promote downstream Pro biosynthesis (Balana-Fouce et al., 2012; Ochocki et al., 2018). Supporting a direct impact of increased Arg catabolism on RASmt B-ALL cell growth, treatment of Reh cells with the arginase inhibitor S-(2-boronoethyl)-L-cysteine (BEC) partially rescued the reduced relative growth rates of the RASmt cells upon reduction of extracellular Arg concentrations (Figures 4G and 4K). Collectively, these results support that that earlier observed differences in Met and Arg levels in cells bearing the KRAS-G12D mutation result from increased Met and Arg catabolism that supports anabolism of polyamines and proline, respectively.

### KRAS-G12D B-ALL cells are sensitive to killing by the polyamine biosynthesis inhibitor DFMO

**Figure 5:**
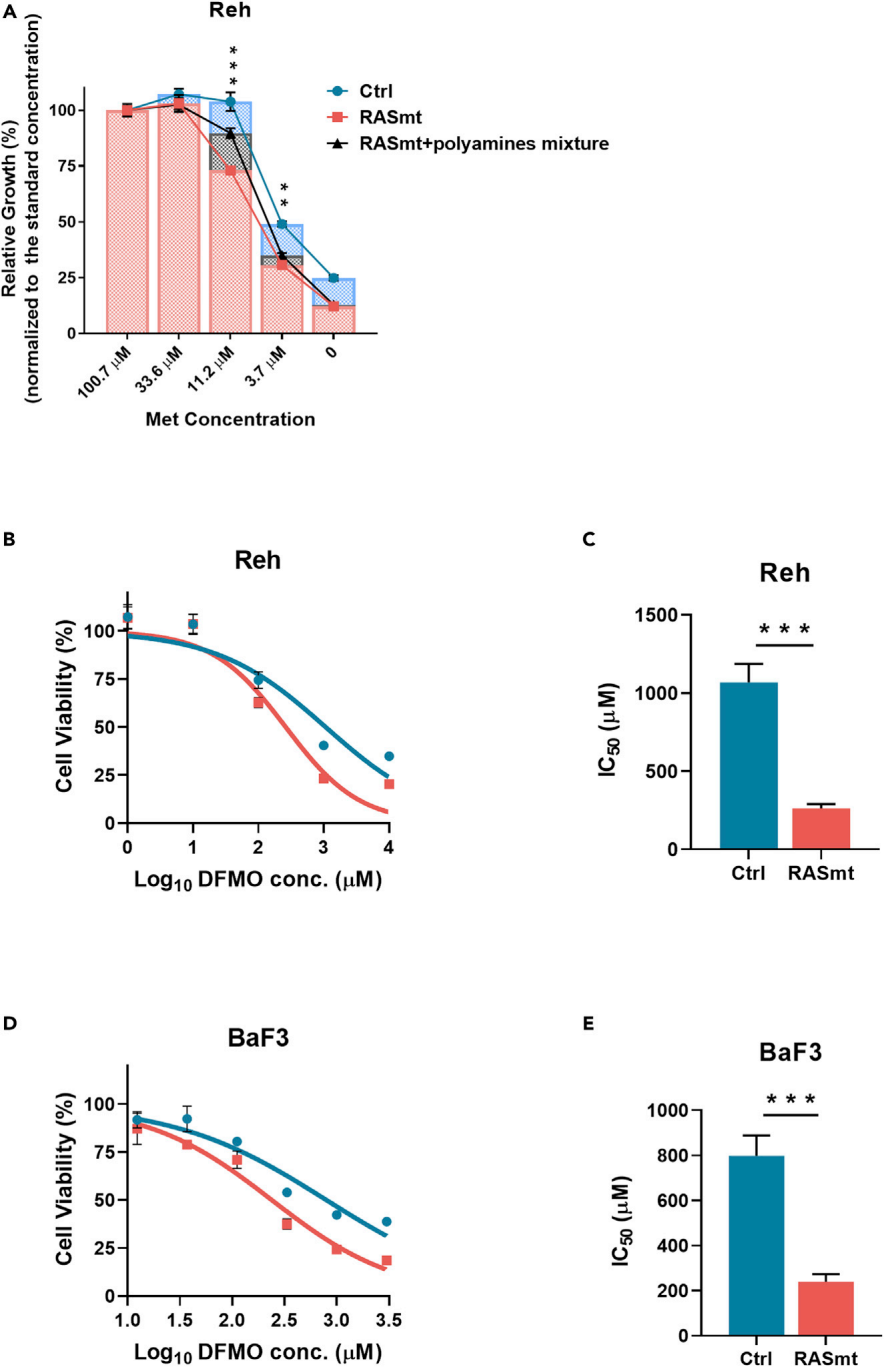
KRAS-G12D B-ALL cells are sensitive to killing by the polyamine biosynthesis inhibitor DFMO (A) The viabilities of various Reh cells supplied with a polyamine mixture (containing 1 μM putrescine, 1 μM spermidine, and 1 μM spermine) grown in media with different concentrations of Met for 72 h. 100.7 μM is the standard concentration of Met in normal RPMI 1640. Data are shown as the mean ± SD ∗∗: p < 0.01, ∗∗∗: p < 0.005 (RASmt vs. RASmt + polyamines); two-tailed Student’s t-tests. (B and C) Viabilities of Ctrl and RASmt Reh cells at increasing concentrations of the polyamine biosynthesis inhibitor DFMO (which targets the ODC1 enzyme) (B) and the IC_50_ values (C). Data are shown as the mean ± SD ∗∗∗: p < 0.005; two-tailed Student’s *t*-tests. (D and E) Viabilities of Ctrl and RASmt BaF3 cells at increasing concentrations of the polyamine biosynthesis inhibitor DFMO (which targets the ODC1 enzyme) (D) and the IC_50_ values (E). BaF3 cells were cultured in media with a low concentration of IL-3 (0.1 ng/mL). Data are shown as the mean ± SD ∗∗∗: p < 0.005; two-tailed Student’s *t*-tests.

We next treated RASmt Reh cells with exogenous polyamines and found that a polyamine mixture (comprising putrescine, spermidine, and spermine) partially rescued the growth of KRAS-G12D mutant cells under Met-limiting conditions (Figure 5A). Further, treatment of Reh and BaF3 cells with difluoromethylornithine (DFMO; an inhibitor of ornithine decarboxylase 1, ODC1) to block polyamine biosynthesis reduced cell viability to a greater extent in RASmt cells than Ctrl cells (Figures 5B and 5D), and the IC_50_ values revealed the significantly greater potency of DFMO against cells bearing the KRAS-G12D mutation (Figures 5C and 5E). These results support that targeting polyamine biosynthesis may represent a promising approach for developing therapeutics to treat KRAS-G12D mutant ALL.

### KRAS-G12D B-ALL cells display activated mTOR signaling and chemical inhibition of mTOR rescues the growth defects of these cells *in vivo*

**Figure 6:**
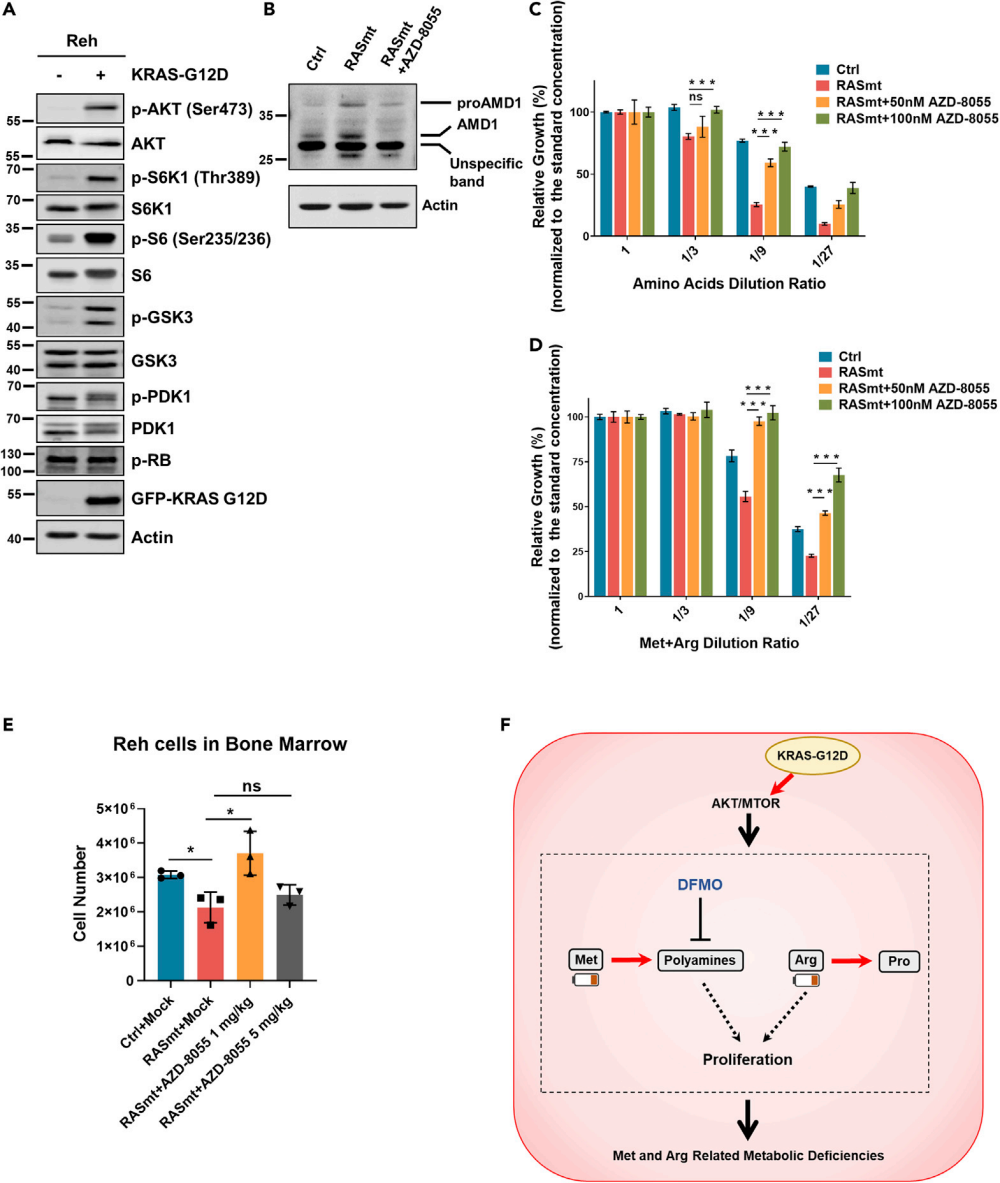
KRAS-G12D B-ALL cells display activated mTOR signaling and chemical inhibition of mTOR rescues the growth defects of these cells *in vivo* (A) The phosphorylation levels of the indicated proteins (AKT, S6K1, S6, and GSK3) in Ctrl and RASmt Reh cells, implicating differential activity in the AKT/mTOR signaling pathways. (B) The protein levels of AMD1 in various Reh cells. Ctrl and RASmt cells were incubated with or without 500 nM AZD-8055 overnight and harvested for blotting. (C) The viabilities of Reh cells pre-treated (or untreated) with the mTOR inhibitor AZD-8055 for 12 h, and then grown in media with different dilution ratios of total 20 amino acids mixture for 72 h. Data are shown as the mean ± SD ns: p > 0.05, ∗∗∗: p < 0.005; two-tailed Student’s *t*-tests. Ctrl: control Reh cells, RASmt: Reh cells expressing KRAS-G12D. (D) The viabilities of Reh cells pre-treated (or untreated) with mTOR inhibitor AZD-8055 for 12 h, and then grown in media with the indicated dilution ratios of a Met/Arg mixture for 72 h. Data are shown as the mean ± SD ∗: p < 0.05, ∗∗: p < 0.01; two-tailed Student’s *t*-tests. (E) The cell numbers of various Reh cells in bone marrow xenografts (of tibias) on the 20^th^ day after tail-injection of 10^7^ Reh cells; mice were treated with the indicated dosage of AZD-8055 twice daily by oral gavage, n = 3. Data are shown as the mean ± SD ns: p > 0.05, ∗: p < 0.05; two-tailed Student’s *t* test. (F) A schematic showing of the KRAS-G12D-induced metabolic vulnerability mechanisms. KRAS-G12D could increase Arg and Met catabolism to support anabolism of Pro and polyamines, which led to reduced intracellular levels of Met and Arg and Met/Arg-related metabolic deficiencies. The ODC1 inhibitor DFMO inhibited polyamine biosynthesis and selectively killed KRAS-mutant cells. See also Figures S4–S6.

Previous studies have shown that increased AKT/mTOR (mammalian target of rapamycin) pathway activity can promote polyamine biosynthesis from Met by stabilizing AMD1 (adenosylmethionine decarboxylase 1) (Zabala-Letona et al., 2017). We therefore examined whether AKT/mTOR signaling is altered in RASmt B-ALL cells grown in normal media. Indeed, monitoring of classic makers including AKT, S6K1, and GSK3 showed significantly higher AKT/mTOR signaling activation in RASmt cells compared to Ctrl cells (Figure 6A). We also observed that the AMD1 protein level was increased in RASmt cells compared to controls and were downregulated by inhibiting mTOR signaling pathway with mTOR kinase inhibitor AZD-8055 in RASmt cells (Figures 6B and S4), a finding consistent with our previous conclusion that the KRAS-G12D mutation somehow promotes the catabolism of Met in support of polyamine biosynthesis in B-ALL cells. Moreover, inhibiting AMD1 with its inhibitor sardomozide (Regenass et al., 1994) also reversed the increased polyamine biosynthesis from Met in RASmt Reh cells (Figure S5)

Finally, *in vitro* experiments with AZD-8055 showed that mTOR signaling inhibition obviously reduced the growth sensitivity of RASmt cells in response to reduced extracellular amino acid concentrations (including total amino acids and specific Arg and Met deficiency) (Figures 6C and 6D). And experiments using Reh cells (including both Ctrl and RASmt cells) in bone marrow xenograft mice showed that chemically inhibiting mTOR signaling by low-dose AZD-8055 could significantly promote the *in vivo* growth of RASmt cells (Figure 6E), but the promoting effect disappeared when we used high-dose AZD-8055 which further decreased the phosphorylation level of AKT in RASmt cells (Figure S6), suggesting a proper mTOR inhibition is important to rescue the growth defects of KRAS-G12D ALL cells. Thus—and recalling our much earlier xenograft experimental finding that RASmt Reh cells grew much more slowly than control Reh cells—our metabolism-based investigation led us from the observation of differential Met levels, to validation of increased polyamine biosynthesis, and ultimately to confirmatory evidence about growth-related impacts of differentially activated mTOR signaling in KRAS-G12D mutation-bearing B-ALL cells.

## Discussion

In the present study, we discovered that the proliferation of B-ALL cells bearing the KRAS-G12D mutation in the bone marrow of xenograft mouse models is strongly impacted by altered amino acid metabolism. Specifically, our analyses revealed that the KRAS-G12D mutation rewires Met and Arg metabolism in B-ALL cells, causing increased Met and Arg catabolism that is accompanied by increased anabolism of polyamines and proline, respectively. After our metabolite rescue and chemical inhibitor studies demonstrated impacts of this metabolic rewiring on B-ALL cell growth, we found that chemically inhibiting the AKT/mTOR pathway can rescue the mutant KRAS-induced amino acid metabolism disruption and promote the *in vivo* growth of KRAS-mutant B-ALL cells (Figure 6F).

Although the capacity of mutant RAS to promote proliferation supports its oncogenic role in malignant transformation, it must be noted that RAS mutations have also been shown to induce proliferation arrest (Bates et al., 1998; Palmero et al., 1998; Serrano et al., 1997); these divergent outcomes depend on cellular context (Guerra et al., 2003; Sarkisian et al., 2007). The KRAS mutation has been shown to promote hyperproliferation in colon cancer and pancreatic cancer (Bryant et al., 2014; Haigis et al., 2008); there are also reports of various mechanisms through which RAS activation can lead to replication stress and cellular senescence (Di Micco et al., 2006; Kotsantis et al., 2016; Maya-Mendoza et al., 2015). Here, we found that the KRAS-G12D mutation rewires amino acid metabolism, so our findings support the notion that activation of RAS mutants is a double-edged sword: it can promote cell proliferation, but it can also induce metabolic vulnerabilities during cancer progression.

Previous studies have suggested that the capacity of B-lineage lymphocytes and ALLs to tolerate oncogenic signaling is limited (Grundschober et al., 2014; Schwartzman et al., 2017; Shojaee et al., 2015), and AKT hyperactivation was shown to inhibit precursor B-ALL development (Shojaee et al., 2016). It therefore seems possible that AKT signaling activity above some threshold level may function to elicit negative selection to remove self-reactive B cells, or perhaps to selectively kill ALL cells (Chen et al., 2015). Based on our observations, hyperactivation of AKT/mTOR signaling promotes the KRAS-G12D mutation-induced rewiring of amino acid metabolism, which compromises the growth of B-ALL cells; this conclusion is supported by our evidence showing that chemically inhibiting mTOR rescued the growth defects of RASmt cells *in vivo*. Further study will be required to ascertain if similar metabolic rewiring may impact oncogenic signaling responses in solid tumors.

Rapidly proliferating tumor cells demand sufficient nutrients supplies (including glucose and amino acids), and limiting nutrient availability can selectively kill tumor cells (Longo and Fontana, 2010; Mihaylova et al., 2014; Vernieri et al., 2016). A previous study of a solid tumor (prostate cancer) showed that mTORC1 activation promotes polyamine biosynthesis by stabilizing its regulating enzyme AMD1 (Zabala-Letona et al., 2017). We also detected that the AMD1 protein level was elevated in RASmt B-ALL cells, and showed that supplementation with polyamines can rescue the observed growth reduction phenotypes of RASmt B-ALL cells under the methionine-limited condition. Polyamines are known to be necessary for cell proliferation (Casero et al., 2018), and polyamine homeostasis was recently implicated in the proliferation of T lymphocytes (Wu et al., 2020). Our results therefore suggest that active polyamine biosynthesis may be required to support the rapid proliferation of KRAS-G12D B-ALLs. Further investigations will be needed to confirm this speculation and to reveal how polyamine metabolism supports the growth of B-ALL cells.

It should be emphasized that an inhibitor (DFMO) of the polyamine biosynthesis enzyme ODC1 has been approved by the US FDA for facial hirsutism treatment, and preclinical data characterized DFMO as a potential tumor prevention reagent (Alexiou et al., 2017). Several DFMO clinical trials are ongoing for solid tumors (www.clinicaltrials.gov, NCT04301843, NCT04696029, NCT00086736, NCT00033371). Our results suggest that DFMO treatment deserves exploration as a potential treatment strategy against KRAS-G12D mutant B-ALL.

### Limitations of the study

The following caveats of the conclusion of this study could be mentioned. Our research focused on B-ALL cell lines expressing exogenous KRAS-G12D, additional models (e.g., knockin cell lines and transgenic mice) would be needed to make the conclusion more universally applicable. Finally, we did not reveal the underlying mechanism of how polyamines and arginine can support KRAS-G12D ALL cells growth, respectively. Further explorations are needed to define the roles of polyamine and arginine in tumor development, which will increase the clinical application value.

## STAR★Methods

### Key resources table

**Table:** 

REAGENT or RESOURCE	SOURCE	IDENTIFIER
**Antibodies**		

phospho-AKT (S473)	Cell Signaling Technology	Cat#4060, RRID: AB_2315049
AKT	Cell Signaling Technology	Cat#2920, RRID: AB_114762
phospho-S6K (T389)	Cell Signaling Technology	Cat#9234, RRID: AB_2269803
phospho-S6 (S235/236)	Cell Signaling Technology	Cat#4858, RRID: AB_916156
S6K	Cell Signaling Technology	Cat#9202, RRID: AB_331676
S6	Cell Signaling Technology	Cat#2317, RRID: AB_2238583
phospho-GSK3 α/β (S9/21)	Cell Signaling Technology	Cat#9331, RRID: AB_329830
GSK3	Cell Signaling Technology	Cat#5676, RRID: AB_10547140
phospho-Rb	Cell Signaling Technology	Cat#8516, RRID: AB_11178658
mme-K	Cell Signaling Technology	Cat#14679, RRID: AB_2798567
dme-K	Cell Signaling Technology	Cat#14117, RRID: AB_2798396
tme-K	Cell Signaling Technology	Cat#14680, RRID: AB_2798568
H3K4-me2	Cell Signaling Technology	Cat#9725, RRID: AB_10205451
H3K4-me3	Cell Signaling Technology	Cat#9751, RRID: AB_2616028
H3K9-me2	Cell Signaling Technology	Cat#4658, RRID: AB_10544405
H3K27-me2	Cell Signaling Technology	Cat#9728, RRID: AB_1281338
H3K27-me3	Cell Signaling Technology	Cat#9733, RRID: AB_2616029
H3K36-me2	Cell Signaling Technology	Cat#2901, RRID: AB_1030983
H3K79-me2	Cell Signaling Technology	Cat#5427, RRID: AB_10693787
phospho-PDK1 (S241)	Abcam	Cat#ab109460, RRID: AB_10866450
PDK1	Abcam	Cat#ab109253, RRID: AB_10861761
RAS	Abcam	Cat#ab52939, RRID: AB_2121042
AMD1	Proteintech	Cat#11052-1-AP, RRID: AB_2226430
Actin	HuaBio	M1210-2
Histone H3	HuaBio	EM30605

**Chemicals, peptides, and recombinant proteins**		

RPMI 1640	Life Technologies	C11875500CP
DMEM	Life Technologies	C11965500CP
Fetal Bovine Serum	Life Technologies	10099141C
Dialyzed Fetal Bovine Serum	Life Technologies	30067334
Glucose-free and amino acid-free RPMI 1640	Life Technologies	Customized
Amino acid-free RPMI 1640	Caisson	RPL22-500ML
Glucose-free RPMI 1640	Life Technologies	11879020
Human plasma-like medium	Life Technologies	A4899101
Glucose solution	Sigma Aldrich	G8769
RPMI 1640 amino acid solution	Sigma Aldrich	R7131
Methionine powder	Sigma Aldrich	M5308
Arginine powder	Sigma Aldrich	A6969
Cysteine powder	Sigma Aldrich	C7352
Murine IL-3	Peprotech	213-13
DFMO	Selleck	S4582
BEC	Selleck	S7929
AZD-8055	Selleck	S1555
Sardomozide dihydrochloride	MedChemExpress	HY-13746B
SBE-β-CD	Selleck	S4592
Captisol	Ligand	-
Polybrene	Sigma Aldrich	TR-1003-G
^13^C_5_-methionine	Cambridge Isotope Laboratories	CLM-893-H-0.05
^13^C_6_-arginine	Cambridge Isotope Laboratories	CLM-2265-H-0.1
^13^C_5_-glutamine	Sigma Aldrich	605166

**Critical commercial assays**		

Fugene 6	Promega	E2691
Annexin V Apoptosis Detection Kit	Thermo Fisher	88-8007-72
CellTiter-Glo Luminescent kits	Promega	G7572
jetPRIME	Polyplus	101000046

**Experimental models: Cell lines**		

Reh	DSMZ	Cat#ACC-22, RRID: CVCL_1650
BaF3	DSMZ	Cat#ACC-300, RRID: CVCL_0161
CCRF-CEM	DSMZ	Cat# ACC-240, RRID: CVCL_0207
KOPN8	DSMZ	Cat# ACC-552, RRID: CVCL_1866
HEK-293T	ATCC, provided by Dr. Ruibao Ren	Cat#CRL-3216, RRID: CVCL_0063
PHE cell	ATCC	Cat#SD-3444, RRID: CVCL_H717

**Experimental models: Organisms/strains**		

Mouse: B-NDG	Biocytogen	110586

**Recombinant DNA**		

MIG empty vector	A gift from Dr. Ruibao Ren	-
MIG-KRAS-G12D	A gift from Dr. Ruibao Ren	-
VSVG	Addgene, provided by Dr. Ruibao Ren	RRID: Addgene_1733
GAG	Addgene, provided by Dr. Ruibao Ren	RRID: Addgene_1732
pECO	Addgene	RRID: Addgene_12371

**Software and algorithms**		

OriginPro	OriginLab	-

### Resource availability

#### Lead contact

Further information and requests for resources and reagents should be directed to and will be fulfilled by the lead contact, Hui Li (lihui@scmc.com.cn)

#### Materials availability

This study did not generate new unique reagents. Any additional resources in this paper are available from the lead contact upon request.

### Experimental model and subject details

#### Mice

Immunodeficient B-NDG mice (NOD-scid strain, female, 6- to 8-week-old) were bought from Biocytogen (China) and were used to generate B-ALL xenograft models in this study. All our animal experiments were approved by the Medical Ethical Committee of Shanghai Jiao Tong University and Shanghai Children’s Medical Center.

#### Cell culture

The following cell lines were used in this study: Reh (human, female, B cell precursor leukemia, ACC-22, DSMZ, RRID: CVCL_1650), BaF3 (mouse, C3H strain, male, pro B cells, ACC-300, DSMZ, RRID: CVCL_0161), CCRF-CEM (human, female, ACC-240, DSMZ, RRID: CVCL_0207), KOPN8 (human, female, T-cell leukemia, ACC-552, DSMZ, RRID: CVCL_1866), HEK-293T (human, female, kidney, CRL-3216, ATCC, RRID: CVCL_0063), PHE cell (human, female, kidney, SD-3444, ATCC, RRID: CVCL_H717). Reh, BaF3, CCRF-CEM and KOPN8 were maintained in RPMI-1640 (Gibco, USA). HEK293T and PHE were cultured in DMEM (Gibco, USA). All media were supplemented with 10% fetal bovine serum (Gibco) and 100 U/mL penicillin and 0.1 mg/mL streptomycin. In addition, medium for BaF3 was supplemented with 10 ng/mL murine IL-3 (Peprotech, USA).

### Method details

#### Generation of the KRAS-G12D mutant Reh cells and BaF3 cells

To generate stable KRAS-G12D mutant cell lines, Reh cells and BaF3 cells were transfected with the retroviral construct MIG-KRAS-G12D. To generate control (Ctrl) cells, Reh cells and BaF3 cells were transfected with the empty retroviral construct. MIG constructs contain GFP coding sequence, both KRAS-G12D cells and the control cells are therefore labeled with GFP.

#### Virus production and transfection

Retroviral constructs for human cell transfection were packaged in plasmids V-SVG and GAG, and transfected into HEK-293T cells using Fugene 6 (Promega, USA); retroviral constructs for murine cell transfection were packaged in plasmid pECO and transfected into PHE cells also using Fugene 6. Virus-containing supernatant was harvested after 48 hr, concentrated in a 100-KD column (Millipore, USA), and transfected into cells supplemented with 8 μg/mL polybrene (Sigma, USA). The medium was changed 24 hr after transfection, and GFP-positive cells were sorted using a Moflo XDP cell sorter (Beckman Coulter, USA).

#### Assessment of cell viability and apoptosis

Cell viability was assessed using CellTiter-Glo Luminescent kits (Promega, USA) according to the manufacturer’s instructions. Apoptosis was assessed by staining with annexin V-Allophycocyanin (APC) and propidium iodide (PI) (Annexin V Apoptosis Detection Kit, Thermo Fisher, USA) followed by flow cytometry on a Canto Ⅱ FACS flow cytometer (BD, USA). All experiments were performed in triplicate.

#### Growth curve analysis

To obtain growth curves for Reh cells grown in normal RPMI 1640 medium, cells were seeded in 96-well plates. Then the cell viability was measured by using CellTiter-Glo Luminescent kits (Promega, USA) at different time points. For growth curves of Reh cells under nutrient-limited conditions, cells were seeded in 96-well plates with RPMI 1640 media containing low concentrations of glucose (1 mM), amino acids (30-fold diluted concentrations of all amino acids) and 10% dialyzed FBS. Then cell viability was measured at different time points. Growth curves of BaF3 cells cultured under nutrient-limited conditions were obtained based on the cell viability of cells cultured in the same medium but also containing a low concentration of IL-3 (0.1 ng/mL). To figure out the growth curves of Reh cells in human plasma-like medium, cells were seeded in 96-well plates with HPLM medium containing 10% dialyzed FBS. Then cell viability was measured at different time points.

#### ALL xenografts

For the Reh xenograft models, Reh cells (1 × 10^7^) were injected into immune-deficient mice B-NDG (Biocytogen, China) through tail-vein injection. For AZD-8055 treatment, AZD-8055 (Selleck, USA) was dissolved in SBE-β-CD (Selleck, USA) or Captisol (Ligand, USA), with administration to mice twice daily by oral gavage starting from the 10^th^ day after tail-injection. The tumor burden of Reh xenografts was determined by the percentage of GFP-labeled Reh cells in total bone marrow cells, which is determined by flow cytometry after removing erythrocytes. All our animal experiments were approved by the Medical Ethical Committee of Shanghai Jiao Tong University and SCMC.

#### Cells sensitivities to concentration changes of glucose and amino acids

To determine cell sensitivities to glucose concentration changes, Reh cells were seeded in 96-well plates, cultured in glucose-free RPMI 1640 medium (Gibco, USA) with a range of glucose concentrations and 10% dialyzed FBS (Gibco, USA) for 72 hr; cell viability was then determined using the CellTiter-Glo Luminescent kits (Promega, USA).

To determine cell sensitivities to amino acid concentration changes, Reh and BaF3 cells were seeded in 96-well plates, cultured in amino acid-free RPMI 1640 medium (Gibco, USA) with various dilution ratios of the RPMI 1640 amino acid mixture (Sigma, USA) and 10% dialyzed FBS for 72 hr, after which cell viability was measured using CellTiter-Glo Luminescent kits. To determine cell sensitivities to Met and Arg concentration changes, cells were seeded in 96-well plates, and were cultured in customized RPMI 1640 medium (Basalmedia, China) with a range of concentrations of certain amino acids (Sigma, USA) and 10% dialyzed FBS for 72 hr, after which cell viability was measured using CellTiter-Glo Luminescent kits.

#### Amino acid consumption assay

To quantify the consumption of amino acids during Reh cell culture, Reh cells were maintained in fresh RPMI 1640 at a density of 1 × 10^6^ cells per mL for 24 hr. After culturing, the medium was collected, and the cells were removed through low-speed centrifugation. The medium was mixed with 50% acetonitrile (Honeywell, USA) and centrifuged at 13,000 ×g for 15 min. The supernatants were then collected and analyzed by LC-MS. Differences between amino acids levels in the medium and those in the control cell-free medium were calculated, thus representing the consumption of each amino acid.

#### Metabolite profiling and isotope tracing

For metabolite profiling, cells were maintained in fresh RPMI 1640 medium at a density of 5 × 10^5^ cells per mL for 2 hr, then cells were harvested and pelleted. To extract metabolites, cells were quenched in cold 80% methanol, and the extracts were centrifuged at 13,000 ×g for 15 min. The supernatants were collected and analyzed by LC-MS as previously described (Wang et al., 2019a).

For isotope tracing, cells were cultured in RPMI-1640 at a density of 5 × 10^5^ cells per mL. After addition of ^13^C_5_-methionine, ^13^C_6_-arginine (Cambridge Isotope Laboratories, USA) or ^13^C_5_-glutamine (Sigma Aldrich, USA), cells were cultured for various durations. To extract metabolites, the cells were quenched in cold 80% methanol, and then extracts were centrifuged at 13,000 ×g for 15 min, and metabolites in the supernatant were analyzed by LC-MS. The culture medium for BaF3 cells contained a low concentration of IL-3 (0.1 ng/mL).

#### Measurement of amino acid levels in bone marrow matrix

Reh cells (1 × 10^7^) were injected into B-NDG mice through tail-vein injection. When the tumor burden reached 25–35% in each xenograft model, the total bone marrow matrix of tibias and femurs were isolated by using equal volume of cold 0.9% NaCl solution. Then, cells were totally removed from the bone marrow matrix solutions through low-speed centrifugation. Amino acids in the supernatant were analyzed by ultra-high-performance liquid chromatography (UHPLC).

#### Western blotting

Cells were harvested in lysis buffer and analyzed by SDS-PAGE with the following antibodies: phospho-AKT (S473), AKT, phospho-S6K (T389), S6K, phospho-S6 (S235/236), S6, phospho-GSK3 (S21), GSK3, phospho-Rb, mme-K, dme-K, tme-K, H3K4-me2, H3K4-me3, H3K9-me2, H3K27-me2, H3K27-me3, H3K36-me2, H3K79-me2 (purchased from Cell Signaling Technology, USA), phospho-PDK1 (S241), PDK1, RAS (bought from Abcam, UK), AMD1 (Proteintech, USA), and Actin (HuaBio, China). Immunoblots were imaged and analyzed using an Odyssey system (LI-COR Biosciences, USA), ImageQuant LAS 4000 (GE Healthcare, USA), and ChemiDoc MP (Biorad, USA).

### Quantification and statistical analysis

Sample sizes and reproducibility for each figure are denoted in the figure captions. All western blots are representative of at least three biologically independent experiments. Statistical significance between conditions was calculated using two-tailed Student’s t-tests, ns: p>0.05, ∗: p<0.05, ∗∗: p<0.01, ∗∗∗: p<0.005. All error bars represent the s.d. All of the statistical details of experiments can be found in the figure legends.

## Data Availability

This paper does not report original code. All data needed to evaluate the conclusions in the paper are present in the paper. Any additional information required to reanalyze the data reported in this paper is available from the lead contact upon request.
