# Supplementary material for: The KRAS-G12D mutation induces metabolic vulnerability in B-cell acute lymphoblastic leukemia
Source: iScience. 2022 Feb 7;25(3):103881. doi: 10.1016/j.isci.2022.103881 (PMC8861657; doi:10.1016/j.isci.2022.103881)
Supplement: Document S1. Figures S1–S6 [file mmc1.pdf]

iScience, Volume *25*

Supplemental information

The KRAS-G12D mutation induces metabolic vulnerability in B-cell acute lymphoblastic leukemia

Yan Xu, Houshun Fang, Yao Chen, Yabin Tang, Huiying Sun, Ziqing Kong, Fan Yang, Renate Kirschner-Schwabe, Liang Zhu, Alex Toker, Ning Xiao, Bin-Bing S. Zhou, and Hui Li

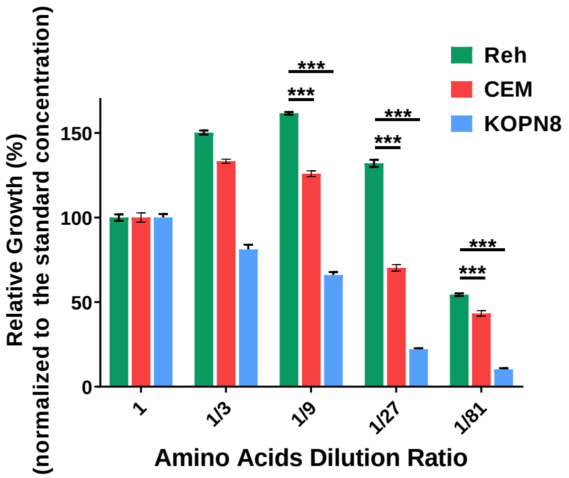

Supplementary Figure 1. The growths of CEM and KOPN8 were more sensitive to altered concentrations of extracellular amino acids than that of Reh cells, Related to Figure 2.

The viabilities of Reh, CEM and KOPN8 cells grown in media with different dilution ratios of a total 20 amino acids mixture for 72 hr. Data are shown as the mean ± s.d. ***: $p<0.005$; two-tailed Student's *t*-tests. Both CEM and KOPN8 cells harbor endogenous KRAS mutations and Reh cells have no mutations in KRAS, NRAS or HRAS.

A.

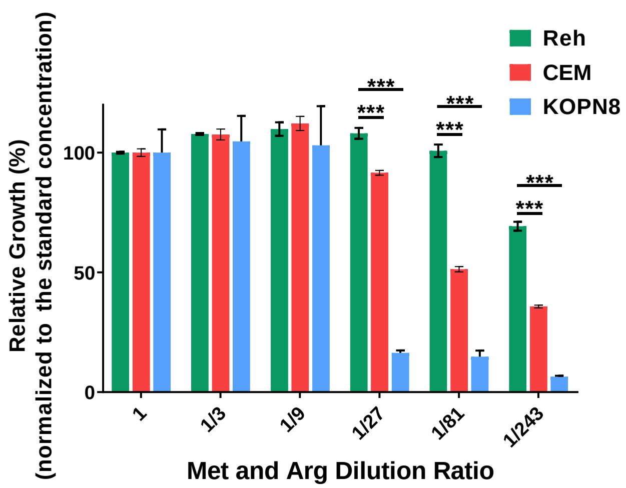

B.

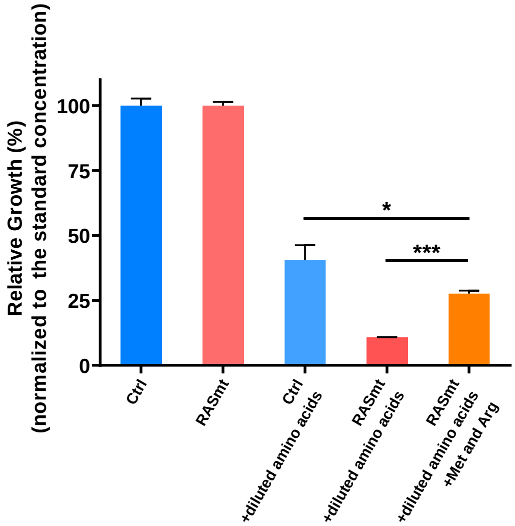

Supplementary Figure 2. The growths of KRAS-mutant cells were more sensitive to altered concentrations of extracellular Met and Arg than that of control cells, Related to Figure 3.

A. The viabilities of Reh, CEM and KOPN8 cells grown in media with different dilution ratios of a Met and Arg mixture for 72 hr. Data are shown as the mean ± s.d. ***: p<0.005; two-tailed Student's *t*-tests.

B. The viabilities of various Reh cells grown in the normal medium or in media with a 27-fold dilution of a total 20 amino acids mixture for 72 hr. For Met and Arg supplementation, a Met and Arg mixture was added into the medium to make the final concentrations of these two amino acids equal to those in normal RPMI 1640. Data are shown as the mean ± s.d. ***: p<0.005; two-tailed Student's *t*-tests.

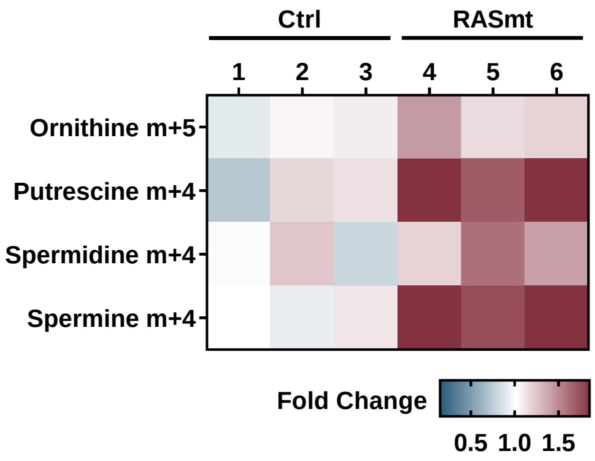

Supplementary Figure 3. A heatmap indicating fold changes in the intracellular levels of labeled metabolites in various Reh cells after $^{13}C_5$-glutamine incubation for 6 hr, Related to Figure 4.

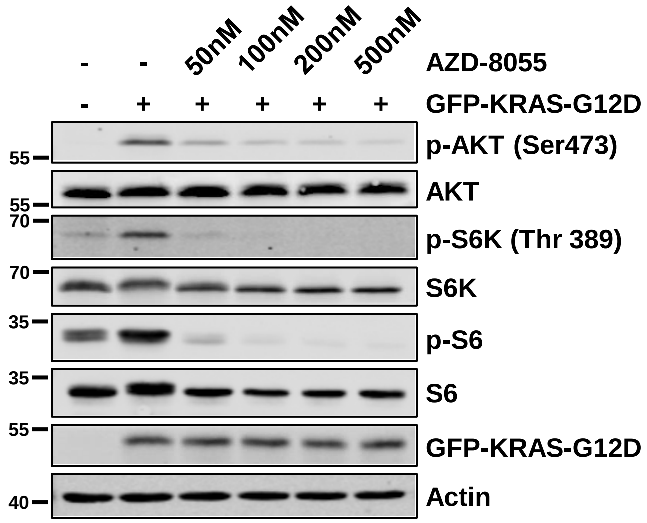

Supplementary Figure 4. The phosphorylation levels of the indicated proteins (AKT, S6K1 and S6) in RASmt Reh cells after incubation with indicated concentrations of AZD-8055 for 12 hr, implicating the inhibitory effect of AZD-8055 on mTOR signaling pathway, Related to Figure 6.

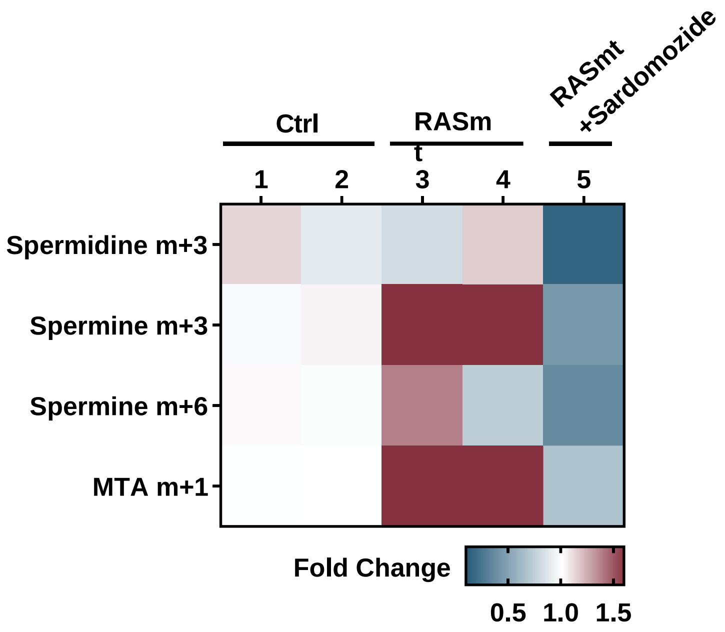

Supplementary Figure 5. A heatmap indicating fold changes in the intracellular levels of labeled metabolites in various Reh cells after $^{13}C_5$-methionine incubation for 4 hr. RASmt cells were pre-treated with or without 1 μM sardomozide for 24 hr before harvest, Related to Figure 6.

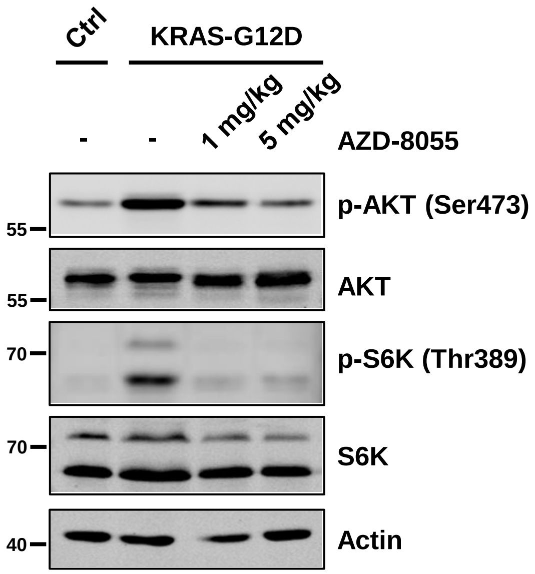

Supplementary Figure 6. The phosphorylation levels of the indicated proteins (AKT and S6K1) in various Reh cells extracted from bone marrow xenografts on the 20th day after tail-injection of 5×10^6 Reh cells; mice were treated with the indicated dosage of AZD-8055 twice daily by oral gavage, Related to Figure 6.
